# Imaging dose in image-guided radiotherapy for localized prostate intensity-modulated radiotherapy: a nationwide survey in Japan

**DOI:** 10.1093/jrr/rraf080

**Published:** 2025-12-18

**Authors:** Satoshi Kito, Takeshi Takizawa, Satoshi Tanabe, Yuhi Suda, Tomomasa Nagahata, Naoki Tohyama, Hiroyuki Okamoto, Takumi Kodama, Yukio Fujita, Hisayuki Miyashita, Kazuya Shinoda, Masahiko Kurooka, Hidetoshi Shimizu, Takeshi Ohno, Masataka Sakamoto

**Affiliations:** Division of Radiation Oncology, Department of Radiology, Tokyo Metropolitan Cancer and Infectious Diseases Center Komagome Hospital, 3-18-22 Honkomagome, Bunkyo-ku, Tokyo 113-8677 Japan; Department of Radiology, Tokyo Metropolitan Bokutoh Hospital, 4-23-15 Kotobashi, Sumida-ku 130-8575 Japan; Department of Radiation Oncology, Niigata Neurosurgical Hospital, 3057 Yamada, Nishi-ku 950-1101 Japan; Department of Radiation Oncology, Niigata University Medical and Dental Hospital, 1-757 Asahimachi-dori, Chuo-ku 951-8510 Japan; Division of Radiation Oncology, Department of Radiology, Tokyo Metropolitan Cancer and Infectious Diseases Center Komagome Hospital, 3-18-22 Honkomagome, Bunkyo-ku, Tokyo 113-8677 Japan; Radiological Division in Osaka Metropolitan University Hospital, 1-5-7 Asahi-chou, Osaka 545-8586 Japan; Department of Radiation Sciences, Komazawa University, 1-23-1 Komazawa, Setagaya, Tokyo 154-8525 Japan; Radiation Safety and Quality Assurance Division, National Cancer Center Hospital, 5-1-1 Tsukiji, Chuo-ku 104-0045 Japan; Department of Radiation Oncology, Saitama Cancer Center, 780, Ooazakomuro, Ina 362-0806 Japan; Department of Radiation Sciences, Komazawa University, 1-23-1 Komazawa, Setagaya, Tokyo 154-8525 Japan; Department of Radiation Oncology, St. Marianna University Hospital, 2-16-1, Sugao, Miyamae-ku, Kawasaki-City, Kanagawa 216-8511 Japan; Department of Radiation Therapy, Ibaraki Prefectural Central Hospital, 6528 Koibuchi, Kasama City, Ibaraki 309-1793 Japan; Department of Radiation Therapy, Tokyo Medical University Hospital, 6-7-1 Nishishinjuku, Shinjuku-ku 160-0023 Japan; Division of Medical Physics, School of Medical Sciences, Fujita Health University, 1-98, Dengakugakubo, Kutsukake, Aichi 470-1192 Japan; Department of Health Sciences, Faculty of Life Sciences, Kumamoto University, 4-24-1 Kuhonji, Chuo-ku, Kumamoto 862-0976 Japan; Department of Radiology, Hamamatsu University School of Medicine, 1-20-1 Handayama, Chuo-ku, Higashi-ku, Hamamatsu, Shizuoka 431-3192 Japan

**Keywords:** image-guided radiotherapy, prostate cancer, diagnostic reference levels, radiological protection, CTDI_vol_, DLP

## Abstract

This study aimed to establish the diagnostic reference levels (DRLs) of imaging doses for image-guided radiotherapy (IGRT) used in intensity-modulated radiotherapy for prostate cancer in Japan. A nationwide survey was conducted to gather data on image acquisition conditions, parameters, and frequencies across 193 radiation therapy institutions using intensity-modulated radiotherapy. IGRT modalities, such as kilovoltage and megavoltage cone-beam computed tomography (CBCT), two-dimensional imaging, and in-room computed tomography (CT), were targeted. Data analysis focused on image acquisition parameters displayed by the devices, such as tube voltage, current, and imaging dose, along with the CT dose index volume (CTDI_vol_) and dose-length product (DLP), were collected from 222 radiotherapy devices. The results showed that kV-CT/CBCT was the most frequently used modality, used in 94% of the institutions. Imaging dose-reduction techniques were adopted by over half of the institutions, with 56% optimizing imaging parameters and 45% reducing the imaging field size or scan length. The 75^th^ percentile for CTDI_vol_ was 16.0 mGy, while that for DLP was 263 mGy·cm, with considerable variation among devices and institutions. This study provides the first large-scale reference data for IGRT imaging doses used for prostate cancer treatment in Japan. These results are critical for improving patient safety by optimizing imaging protocols and establishing DRLs tailored to IGRT. These findings will serve as a basis for further refinement of radiological protection practices in Japan.

## INTRODUCTION

Image registration for a patient set-up is an indispensable technique for irradiating a target with the treatment beam in radiotherapy. Image-guided radiotherapy (IGRT) using X-rays employs techniques, such as the megavoltage electronic portal imaging device (MV-EPID), MV computed tomography (MVCT), and cone-beam CT (MV-CBCT), kilovoltage two-dimensional (kV-2D) imaging, kV-CBCT, and in-room CT mounted to the linear accelerator (LINAC) [1–4]. In IGRT, the isocenter is aligned with the center of the image before treatment. If unexpected movement is detected during treatment, the session is temporarily interrupted, and image registration is repeated to correct the position error. The combined use of IGRT with technologies, such as intensity-modulated radiotherapy (IMRT) and stereotactic body radiotherapy, has allowed for a further concentration of the dose at the target and a reduction in the dose delivered to healthy tissues. Additionally, it adjusts to changes in the patient’s body shape during treatment, allowing adaptive radiotherapy using images acquired during IGRT [5–7].

Patients are exposed to a significant number of X-rays to acquire IGRT images. Unlike diagnostic imaging procedures, IGRT image acquisition is repeated daily, and the irradiation area is larger than the treatment volume. As radiation to unwanted areas increases the risk of secondary cancer [8], the International Commission on Radiological Protection recommends the use of medical procedures and optimal radiological protection to manage patient radiation doses and avoid unnecessary radiation exposure during medical imaging [9]. In recent years, diagnostic reference levels (DRLs) for imaging diagnoses have been reported in Japan [10–12]. DRLs are established as indicators for optimizing radiation protection in medical exposure. They provide a reference point for individual institutions to assess the appropriateness of their radiation doses and to guide improvement efforts. IEC60601–2-44 [13] requires the display of CT dose metrics, such as the CT dose index volume (CTDI_vol_) and dose-length product (DLP) [14, 15], as proof of mechanical performance. In the concept of DRLs, emphasis is placed on quantities that are easy to collect and evaluate over time at all institutions, even if they lack accuracy. In selecting this quantity, the displayed value of CTDI_vol_ of the device, which is evaluated in conjunction with imaging conditions such as the mAs, serves as an effective index for each institution to be aware of their radiation exposure dose.

The ICRP-135 states that “DRLs are not intended for use in radiation therapy, but they should be considered for imaging for treatment planning, treatment rehearsal, and patient set-up verification in radiotherapy [9].” In Japan, Kito *et al*. [16] reported imaging doses for planning CT scan protocols to establish DRLs in 2023. Then, Japan DRLs 2025 included radiotherapy planning CT protocols [12]. In addition, Okamoto *et al*. [17] reported the imaging dose related to brachytherapy. Several small-scale studies have provided reference data for establishing DRLs for imaging doses associated with commonly used IGRT devices [18–21]. Sakai et al. reported data on CBCT from nine different devices [18]. However, the DRLs for the imaging dose for IGRT have not yet been determined in Japan.

AAPM TG-75 [22] and TG-180 [23] are essential guidelines for IGRT imaging doses. TG-180 categorizes imaging modalities into MV-2D EPID, kV-2D imaging, kV-CBCT, and MV-CBCT, providing estimated imaging doses for various at-risk organs. Additionally, it summarizes the imaging parameters and doses for devices commonly used at the time of publication, with results organized by manufacturer. However, further investigation is required regarding the devices and information not covered in TG-180. Collecting imaging dose data can also be challenging because of the differences in IGRT devices used at each institution.

To establish DRLs for IGRT imaging doses using X-rays in Japan, this study conducted a nationwide survey focusing on image acquisition conditions, parameters, and frequencies in institutions that perform IMRT for localized prostate cancer. Additionally, for CT and CBCT systems, the CTDI_vol_ and DLP values displayed by the devices were aggregated.

## MATERIALS AND METHODS

### Questionnaire summary

A survey was created using Excel (Microsoft, Redmond, WA, USA) and distributed through the mailing list of the Japan Society for Radiation Oncology (JASTRO). This study was approved by the Ethics Committee of the Tokyo Metropolitan Bokutoh Hospital (IRB02–097). Conducted with the cooperation of JASTRO, the Measurement Committee, the Diagnostic Radiology Subcommittee, and the Radiation Protection Committee of the Japan Society of Medical Physics, the survey was carried out from 21 September to 25 November 2022, focusing on radiotherapy institutions in Japan. The collected data targeted positional verification of prostate IMRT at each institution. The main items of the survey included dose-fractionation methods for prostate IMRT, positional verification protocols (methods and imaging conditions), CTDI_vol_ and DLP in CT/CBCT systems, methods for reducing imaging doses, methods for recording imaging conditions, and the frequency of quality control of imaging doses. A summary of the key questions is provided in Table 1. The imaging conditions and doses were limited to adult patients weighing 40–80 kg [16], and the representative values used at each institution were collected.

**Table 1:** Questionnaire summary of basic information in each modality with image-guided radiotherapy for patients with prostate cancer receiving intensity-modulated radiotherapy

Property	Answer
Dose fractionation	Conventional fractionation (1.8–2.0 Gy/fr)
	Moderate hypofractionation (2.4–3.4 Gy/fr)
	Ultra hypofractionation (> 5.0 Gy/fr)
IGRT techniques	kV-2D
	MV-2D
	kV-CT/CBCT
	MV-CT/CBCT
	kV-2D and kV-CT/CBCT
	MV-2D and MV-CT/CBCT
	Others^a^
Number of image acquisitions	Image acquisitions per fraction
	Image acquisitions per total fraction
Imaging parameters of kV-2D technique	Tube voltage (kV)
	Tube current (mAs) per image
	X-ray directions (frontal/lateral/diagonal direction)
Imaging parameters of kV-CT/CBCT technique	Tube voltage (kV)
	Tube current (mAs)
	Scan length (cm)
	Scan angle (full/partial scan)
Imaging doses of kV-CT/CBCT technique	CTDI_vol_ (mGy)
	DLP (mGy･cm)
	Total CTDI_vol_ (mGy)
	Total DLP (mGy･cm)
Available techniques to reduce imaging dose to patients	Reduce the imaging field size or scan length
	Reduce the image acquisition
	Consideration of imaging direction
	Consideration of imaging parameters
	Use of radiation-free image-guided techniques
	nothing in particular
	Others
Recording methods of imaging parameters	Automatic recording through communication with information systems such as RIS
	Manual recording on information systems such as RIS
	Manual recording on spreadsheet and database software
	Recording by capturing the imaging parameters displayed on the operating screen
	Handwritten records
	Not recorded
	others
Frequency of quality control on imaging doses	Daily
	Weekly
	Monthly
	Semi-annually
	Annually
	Timing of machine installation and maintenance
	Not checked

*Abbreviations*: kV-2D, kilovoltage two-dimensional imaging; MV-2D, megavoltage two-dimensional imaging; kV-CT/CBCT, kilovoltage computed tomography/cone-beam computed tomography imaging; MV-CT/CBCT, megavoltage computed tomography/cone-beam computed tomography imaging; CTDI_vol_, computed tomography dose index volume; DLP, dose-length product; RIS, Radiology information system.

^a^Combined use of IGRT modalities including radiation-free techniques

### Data analysis

Data items in IGRT modalities with five or more responses were included in the analysis. Responses regarding the prescribed dose were classified into three groups: conventional fractionation, moderate hypofractionation, and ultra-hypofractionation (Table 1) based on guidelines from the American Society for Radiation Oncology, the American Society of Clinical Oncology, and the American Urological Association [24]. The number of imaging sessions was calculated for each fraction and for the total treatment period across the different IGRT modalities (kV-2D, MV-2D, kV-CT/CBCT, and MV-CT/CBCT) for each fractionation group. The imaging conditions were aggregated using the IGRT modality.

For kV-2D imaging, the medians of the kV and mAs values for each imaging direction (anteroposterior, lateral, and oblique) were calculated. Images in the anteroposterior and lateral directions included those taken using LINAC-integrated kV-2D devices, excluding CyberKnife (Accuray, Sunnyvale, CA, USA), whereas oblique imaging included LINAC-integrated kV-2D devices (CK), ExacTrac (Brainlab, Munich, Germany), and SyncTraX FX4 (Shimadzu, Kyoto, Japan). Owing to the differences in imaging mechanisms, the CT and CBCT imaging conditions were treated separately.

For kV-CBCT imaging, the median of the tube voltage (kV), tube current (mAs), and scan length (cm) per scan angle (full or partial scan) were calculated. In this study, CTDI_vol_ displayed by the devices was used for analysis. Although the CTDI notation varies among manufacturers, it is synonymous with CTDI_vol_. Therefore, CTDI_vol_ was uniformly used in this analysis. The product of the dose per imaging session and the total number of imaging sessions over the treatment period was defined as the cumulative dose (Total CTDI_vol_, Total DLP) for the entire treatment period. The medians, along with the 25th and 75th percentiles, of CTDI_vol_ and DLP were calculated for each CT and CBCT session; for CBCT, these were further stratified by the scan angle. Total CTDI_vol_ and Total DLP were calculated separately for each fractionation category.

## RESULTS

### Questionnaire summary

Responses regarding 222 radiotherapy devices were obtained from 193 institutions. According to the 2020 Survey of Medical Facilities by the Ministry of Health, Labour and Welfare [25], 424 institutions in Japan were equipped with IMRT or other advanced radiotherapy capabilities. The proportion of valid responses was 46% calculated relative to this total. The number of responses regarding each device manufacturer was 121 for Varian Medical Systems (Varian, Palo Alto, CA, USA) (54.5%), 50 for Elekta (Elekta, Stockholm, Sweden) (22.5%), 6 for Siemens (Siemens Healthineers, Erlangen, Germany) (2.7%), 28 for TomoTherapy (Accuray, Sunnyvale, CA, USA) (12.6%), 11 for CK (5%), and 6 for Vero (MHI-TM2000, Mitsubishi Heavy Industries, Tokyo, Japan) (2.7%). The breakdown according to dose fractionation was 118 for conventional fractionation (53.2%), 90 for moderate hypofractionation (40.5%), and 14 for ultra-hypofractionation (6.3%). Ultra-hypofractionation was used in all CK institutions. The breakdown of the IGRT techniques used was as follows: kV-CT/CBCT: 114 (51.4%), kV-2D and kV-CT/CBCT: 67 (30.2%), MV-CT/CBCT: 22 (9.9%), kV-2D: 12 (5.4%), kV-CT/CBCT and radiation-free techniques: 5 (2.3%), MV-2D and MV-CT/CBCT: 1 (0.5%), and MV-2D: 1 (0.5%).

The responses regarding efforts to manage the imaging dose in each institution are shown in Table 2. The most commonly adopted measures for dose reduction were consideration of imaging parameters (56%), followed by reduction of imaging field size (45%). For recording methods of imaging parameters, most were not recorded (39%), followed by manual recording on information systems, such as radiology information system (RIS) (23%), and automatic recording through communication with information systems, such as RIS (16%). For frequency of quality control on imaging doses, the most common practice was ‘Timing of machine installation and maintenance’ (33%), followed by “Not checked” (26%) and “Annually” (23%).

**Table 2:** Survey and responses regarding efforts to manage imaging dose in each institution

Questions and options	N (%)^a^
Available techniques to reduce imaging dose to patients (single choice)	
・Reduce the imaging field size	99 (44.6)
・Reduce the image acquisition	19 (8.6)
・Consideration of imaging direction	34 (15.3)
・Consideration of imaging parameters	124 (55.9)
・Use of radiation-free image-guided techniques	4 (1.8)
・Nothing in particular	31 (14.0)
・Others	16 (7.2)
Recording methods of imaging parameters (multi-choice)	
・Automatic recording through communication with information systems such as RIS	35 (15.8)
・Manual recording on information systems such as RIS	51 (23.0)
・Manual recording on spreadsheet and database software	17 (7.7)
・Recording by capturing the imaging parameters displayed on the operating screen	10 (4.5)
・Handwritten records	7 (3.2)
・Not recorded	87 (39.2)
・Others	15 (6.8)
Frequency of quality control on imaging doses (multi-choice)	
・Daily	5 (2.3)
・Weekly	1 (0.5)
・Monthly	22 (9.9)
・Semi-annually	11 (5.0)
・Annually	51 (23.0)
・Timing of machine installation and maintenance	74 (33.3)
・Not checked	58 (26.1)

*Abbreviations*: RIS = Radiology information system; N, number of sample size.

^a^The numbers in parentheses are the percentage of the total.

### Number of image acquisitions

The number of images acquired for each modality is listed in Table 3. The medians of total number of image acquisitions (per fraction) in the conventional fractionation and moderate hypofractionation groups, respectively, were: kV-2D, excluding CK: 78 (2) and 54 (2); kV-CT/CBCT: 38 (1) and 20 (1); and MV-CT/CBCT: 39 (1) and 21 (1). The value for kV-2D, CK was 489 (98). As the number of responses for the MV-2D modality was less than five, it was excluded from the analysis.

**Table 3:** Number of image acquisitions in each modality with image-guided radiotherapy for patients with prostate cancer receiving intensity-modulated radiotherapy

Modality	Dose fractionation (median of fraction numbers)	N (%)^a^	Treatment fraction	Number of image acquisitions^b^
kV-2D	CF (38)	37 (57.8)	Per fraction	2.0 (0.9–4.2)
			Total fraction	78 (34–160)
	M-HF (20)	27 (42.2)	Per fraction	2.0 (1.0–6.0)
			Total fraction	54 (20–168)
kV-CT/CBCT	CF (38)	104 (56.8)	Per fraction	1.0 (0.2–1.2)
			Total fraction	38 (7–46)
	M-HF (20)	76 (41.5)	Per fraction	1.0 (0.3–2.0)
			Total fraction	20 (6–51)
MV-CT/CBCT	CF (39)	10 (43.5)	Per fraction	1.0 (1.0–1.0)
			Total fraction	39 (34–39)
	M-HF (20)	13 (56.5)	Per fraction	1.0 (1.0–1.2)
			Total fraction	21 (20–33)

*Abbreviations*: kV-2D, kilovoltage two-dimensional imaging; kV-CT/CBCT, kilovoltage computed tomography/cone-beam computed tomography imaging; MV-CT/CBCT, megavoltage computed tomography/cone-beam computed tomography imaging; CF, conventional fractionation; M-HF, moderate hypofractionation; N, number of sample size.

^a^The numbers in parentheses are the percentage of the total. Fraction categories with fewer than five responses were excluded from the table.

^b^Data are shown as the median (minimum – maximum).

### Imaging parameters

For kV-2D imaging, the median tube voltages for frontal, lateral, and diagonal projections were 75, 100, and 120 kV, respectively (Table 4). The tube currents (per image) were 5.0, 15.0, and 12.5 mAs.

**Table 4:** Imaging parameters of kV-2D modality

Direction	N (%)^a^	Tube voltage (kV)^b^	Tube current per scan (mAs)^b^
Frontal	29 (26.4)	75 (70–120)	5.0 (1.3–25.0)
Lateral	30 (27.3)	100 (85–140)	15.0 (2.0–80.0)
Diagonal	51 (46.4)	120 (80–135)	12.5 (1.6–102.4)

*Abbreviations*: N, number of sample size.

^a^The numbers in parentheses are the percentage of the total.

^b^Data are shown as the median (minimum – maximum).

For kV-CBCT imaging, the median tube voltage and scan length were 125 kV and 17.5 cm, respectively, with tube currents of 672 mAs for full scans and 500 mAs for partial scans (Table 5). For the kV-CBCT system associated with the Elekta LINACs, the scan length (cm) was calculated based on the reported collimator cassette settings. As the majority of responses were from TomoTherapy users for kV-CT and MV-CT, data based on TomoTherapy-specific imaging conditions were compiled (Supplementary Tables 1 and 2). The kV-CT using in-room CT was excluded from the analysis because the number of responses was fewer than five.

**Table 5:** Imaging parameters of kV-CBCT modality

Scan angle	N (%)	Tube voltage (kV)^a^	Tube current per scan (mAs)^b^	Scan length (cm)^b^
Full scan	57 (36.1)	125 (72–125)	672 (25–1690)	17.5 (10.0–30.0)
Partial scan	101 (63.9)	125 (70–125)	500 (37–1139)	17.5 (10.0–27.7)

*Abbreviations*: N, number of sample size.

^a^The numbers in parentheses are the percentage of the total.

^b^Data are shown as the median (minimum – maximum).

### Imaging dose

**Fig. 1:**
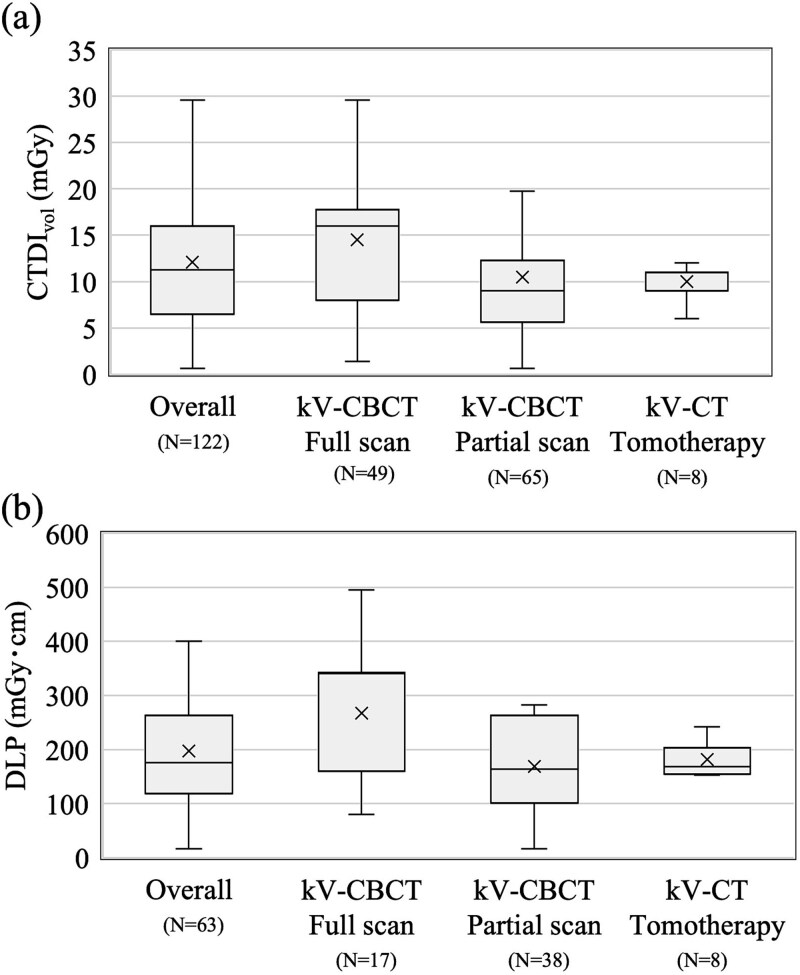
Box plots of imaging dose in kV-CT/CBCT for overall and each modality: (a) CTDI_vol_, (b) DLP. The box represents the interquartile range, and the whiskers indicate the minimum and maximum values within a specific range.

**Fig. 2:**
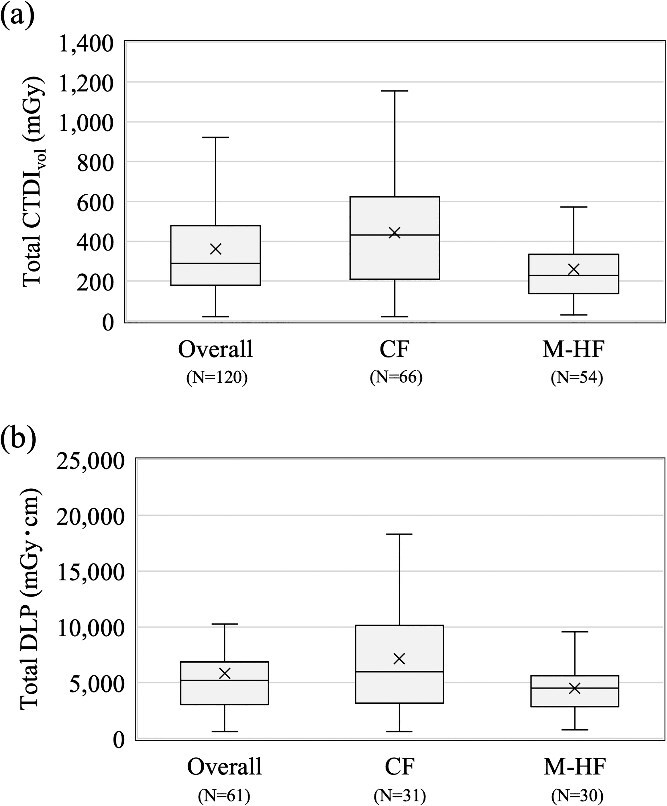
Box plots of total imaging dose in kV-CT/CBCT for overall and each dose fractionation: (a) Total CTDI_vol_, (b) Total DLP. The interpretation of this diagram follows that of Fig. 1.

The results for CTDI_vol_ and DLP, as well as those for Total CTDI_vol_ and Total DLP in kV-CT/CBCT imaging, are shown in Figs. 1 and 2, respectively. The CTDI_vol_ and DLP were distinguished among kV-CBCT full-scan, kV-CBCT partial scan, and kV-CT (TomoTherapy), and the combined results are presented as the overall group. For the total CTDI_vol_ and DLP, the data were distinguished between conventional and moderate hypofractionation; the combined results are presented as the overall group. For CTDI_vol_, the 75^th^ percentiles (50th percentiles) for the overall group, kV-CBCT full and partial scan, and kV-CT (TomoTherapy) were 16.0 (11.3), 17.8 (16.0), 12.3 (9.0), and 11.0 (11.0) mGy, respectively; the 75^th^ percentiles (50th percentiles) of DLP were 263 (176), 342 (340), 263 (164), and 204 (169) mGy·cm, respectively. For Total CTDI_vol_, the 75th percentiles (50th percentiles) for the overall, conventional fractionation, and moderate hypofractionation groups were 480 (289), 624 (431), and 335 (228) mGy, respectively; the 75th percentiles (50th percentiles) for Total DLP were 6863 (5195), 10 129 (5967), and 5619 (4498) mGy·cm, respectively. Data for kV-CT using in-room CT and MV-CT/CBCT were excluded from the analyses of CTDI_vol_ and DLP because they had fewer than five responses; data from the ultra-hypofractionation group were excluded from the analyses of Total CTDI_vol_ and Total DLP for the same reason. The median CTDI_vol_ for vendor-specific systems with five or more CTDI_vol_ responses ranged from 11.0 to 11.7 mGy (Supplementary Table 3 and Fig. 1).

## DISCUSSION

To the best of our knowledge, this is the first nationwide survey of imaging conditions, parameters, and frequency for each IGRT device used in prostate IMRT across various institutions. The response rate in this survey was 46%, which is comparable to the rates reported in the domestic DRLs 2025 [12] surveys for adult diagnostic CT (13.7%) and treatment planning CT (43%). Furthermore, we consider this response rate to be indicative of the current state in Japan, reflecting the existing lack of sufficient guidelines and consensus regarding the measurement and reporting of IGRT imaging doses; thus, the response rate serves as an indicator of the level of interest in IGRT dose. The results included data from 193 institutions and 222 devices, with 94% of institutions using CBCT. In the 2016 ASTRO survey, 76.6% of patients were treated with CBCT or MVCT for intact prostate IMRT [26]. A Japanese national survey in 2019 reported that the prevalence of kV-CBCT+MV-CBCT in prostate IMRT was 82.7% [27]. This difference in usage rates suggests an increase in the use of CBCT over time. Regardless of dose fractionation, the median number of image acquisitions was once for CT/CBCT and twice for kV-2D (Table 3). In the ASTRO survey, 96% of the institutions performed daily IGRT for intact prostate IMRT, which is consistent with the results of this study. More than half of institutions (57.7%) performed partial scans for kV CT/CBCT imaging. This result is likely related to the findings in Table 2, where 55.9% considered “imaging parameters” and 15.3% considered “imaging direction”.

For the kV-CT/CBCT imaging dose, the CTDI_vol_ and DLP were evaluated per fraction and in total. Sakai et al. assessed the weighted cone-beam dose index and reported values ranging from 1.47 to 20.9 mGy for pelvic protocols [18]; these values fall within the range of the CTDI_vol_ observed in this study (0.6–29.6 mGy). Unlike diagnostic radiology, however, radiation therapy requires multiple imaging sessions, making it important to evaluate cumulative values [19]; to assess this, the total DLP was calculated for each fractionation schedule and scanning range. According to AAPM TG 180 [23], it is necessary to manage exposure doses to be as low as reasonably achievable; if the target exposure dose in IGRT exceeds 5% of the prescribed dose, it should be considered when creating a treatment plan. The exposure dose at all institutions was within 5% of the prescribed dose, indicating that there was no immediate need for any institution to adjust its imaging dose in its treatment planning (Fig. 2).

The median image acquisitions per fraction for kV-2D (CF and M-HF) was 2.0. However, the M-HF median total acquisition was 54, thereby exceeding the nominal product of 20 fractions × 2.0 acquisitions per fraction. This discrepancy is attributable to the use of 28-fraction schedules at some institutions within the M-HF group, deviating from the median of 20 fractions. In contrast, kV-CT/CBCT and MV-CT/CBCT showed a median of 1.0 acquisition per fraction, with no inconsistency noted in the total number of fractions over the full treatment course. While the inconsistent application of IGRT imaging parameters across fractions may have caused the total CTDI_vol_ calculated in this survey to deviate from actual values, the errors are unlikely to be significant because the data are based on representative institutional settings.

Regarding efforts toward managing the imaging dose, 14% of the institutions did not devise methods to reduce the exposure dose (Table 2), and the total percentage of institutions that recorded irradiation conditions related to imaging guidance was low (60.8%). Recording the irradiation conditions for image guidance is critical to properly control the exposure dose administered to all patients. In addition, 59.4% of the institutions did not conduct quality control (QC) of IGRT devices or performed QC only at the time of device installation, which is insufficient. We hope that this study will raise awareness regarding the irradiation conditions and QC for IGRT devices.

The CTDI_vol_ and DLP values obtained in this study could serve as valuable data not only for institutions performing prostate IMRT but also for those conducting three-dimensional conformal radiotherapy. If the CTDI_vol_ or DLP values at any institution are significantly higher than those obtained in this study, the imaging conditions and related processes should be reviewed. However, it is important to avoid reducing the imaging dose excessively without considering its impact on the quality of the verified images.

This study had several limitations. It has not been possible to confirm whether the CTDI_vol_ reported by each institution is truly appropriate. The data presented in this study were not subdivided according to the type of treatment equipment used; as more than half of the responses were regarding a single manufacturer, there may have been selection bias in the obtained figures. However, it is challenging to interpret the small number of data points (*n* < 5) obtained by subdividing them by the type of treatment equipment. Nevertheless, the findings of this study contribute to the review of imaging conditions, frequency, and dose for each positioning method in prostate IMRT, leading to more appropriate radiation dose management. These findings can serve as a foundation for establishing DRLs based on exposure doses during IGRT.

## Supplementary Material

8R_Supplemental_rev1_clean_v20251001a_rraf080

## References

[1] Murphy MJ . Tracking moving organs in real time. Semin Radiat Oncol 2004;14:91–100. 10.1053/j.semradonc.2003.10.005. 14752737

[2] Mageras GS . Introduction: management of target localization uncertainties in external-beam therapy. Semin Radiat Oncol 2005;15:133–5. 10.1016/j.semradonc.2005.01.008. 15983938

[3] Jaffray DA . Emergent technologies for 3-dimensional image-guided radiation delivery. Semin Radiat Oncol 2005;15:208–16. 10.1016/j.semradonc.2005.01.003. 15983946

[4] Xing L, Thorndyke B, Schreibmann E et al. Overview of image-guided radiation therapy. Med Dosim 2006;31:91–112. 10.1016/j.meddos.2005.12.004. 16690451

[5] Green OL, Henke LE, Hugo GD. Practical clinical workflows for online and offline adaptive radiation therapy. Semin Radiat Oncol 2019;29:219–27. 10.1016/j.semradonc.2019.02.004. 31027639 PMC6487881

[6] Xing L, Siebers J, Keall P. Computational challenges for image-guided radiation therapy: framework and current research. Semin Radiat Oncol 2007;17:245–57. 10.1016/j.semradonc.2007.07.004. 17903702

[7] Castadot P, Lee JA, Geets X, Grégoire V. Adaptive radiotherapy of head and neck cancer. Semin Radiat Oncol 2010;20:84–93. 10.1016/j.semradonc.2009.11.002. 20219546

[8] Aird EG . Second cancer risk, concomitant exposures and IRMER(2000). Br J Radiol 2004;77:983–5. 10.1259/bjr/56613233. 15569638

[9] Vañó E, Miller DL, Martin CJ et al. ICRP publication 135: diagnostic reference levels in medical imaging. Ann ICRP 2017;46:1–144. 10.1177/0146645317717209. 29065694

[10] Japan Network for Research and Information on Medical Exposure (J-RIME) , National Diagnostic Reference Levels in Japan. 2015, - Japan DRLs 2015-, https://j-rime.qst.go.jp/report/DRLhoukokusyoEng.pdf. Accessed 2025 Sep 25.

[11] Japan Network for Research and Information on Medical Exposure (J-RIME) . National Diagnostic Reference Levels in Japan. 2020, - Japan DRLs 2020-, https://j-rime.qst.go.jp/report/DRL2020_Engver.pdf. Accessed 2025 Sep 25.

[12] Japan Network for Research and Information on Medical Exposure (J-RIME) . National Diagnostic Reference Levels in Japan. 2025, - Japan DRLs 2025-, 2025-07, https://j-rime.qst.go.jp/report/JapanDRLs2025_en.pdf. Accessed 2025 Sep 25.

[13] IEC 60601–2-44 Medical Electrical Equipment-Part 2–44. Particular Requirements for the Basic Safety and Essential Performance of X-Ray Equipment for Computed Tomography, 3rd edn. International Electrotechnical Commission, 2009.

[14] ICRP . Managing patient dose in computed tomography. ICRP Publication 87. Ann. ICRP 2000;30:7. 10.1016/S0146-6453(01)00049-5. 11711158

[15] Dixon RL, Anderson JA, Bakalyar DM et al. Comprehensive Methodology for the Evaluation of Radiation Dose in X-Ray Computed Tomography, AAPM Task Group 111, Report of AAPM Task Group 111: The Future of CT Dosimetry. MD: American Association of Physicists in Medicine College Park, 2010.

[16] Kito S, Suda Y, Tanabe S et al. Radiological imaging protection: a study on imaging dose used while planning computed tomography for external radiotherapy in Japan. J Radiat Res 2024;65:159–67. 10.1093/jrr/rrad098. 38151953 PMC10959444

[17] Okamoto H, Kito S, Tohyama N et al. Radiation protection in radiological imaging: a survey of imaging modalities used in Japanese institutions for verifying applicator placements in high-dose-rate brachytherapy. J Radiat Res 2021;62:58–66. 10.1093/jrr/rraa088. 33074329 PMC7779356

[18] Sakai Y, Monzen H, Takei Y et al. Evaluation of in-room volumetric imaging doses for image-guided radiotherapy: a multi-institutional study. J Med Phys 2023;48:189–94. 10.4103/jmp.jmp_109_22. 37576099 PMC10419753

[19] Stock M, Palm A, Altendorfer A et al. IGRT induced dose burden for a variety of imaging protocols at two different anatomical sites. Radiother Oncol 2012;102:355–63. 10.1016/j.radonc.2011.10.005. 22098793

[20] Martin CJ, Abuhaimed A. Variations in size-specific effective dose with patient stature and beam width for kV cone beam CT imaging in radiotherapy. J Radiol Prot 2022;42:031512. 10.1088/1361-6498/ac85fa. 35917802

[21] Batumalai V, Holloway LC, Kumar S et al. Survey of image-guided radiotherapy use in Australia. J Med Imaging Radiat Oncol 2017;61:394–401. 10.1111/1754-9485.12556. 27863010

[22] Murphy MJ, Balter J, Balter S et al. The management of imaging dose during image-guided radiotherapy: report of the AAPM task group 75. Med Phys 2007;34:4041–63. 10.1118/1.2775667. 17985650

[23] Ding GX, Alaei P, Curran B et al. Image guidance doses delivered during radiotherapy: quantification, management, and reduction: report of the AAPM therapy physics committee task group 180. Med Phys 2018;45:e84–99. 10.1002/mp.12824. 29468678

[24] Morgan SC, Hoffman K, Loblaw DA et al. Hypofractionated radiation therapy for localized prostate cancer: executive summary of an ASTRO, ASCO, and AUA evidence-based guideline. Pract Radiat Oncol 2018;8:354–60. 10.1016/j.prro.2018.08.002. 30322661

[25] Japanese Ministry of Health, Labour and Welfare . Survey of Medical Facilities. 2020, Table T75: Number of institutions with IMRT and other advanced radiotherapy facilities. e-Stat. Published 2023. Available from: https://www.e-stat.go.jp/dbview?sid=0004002037. Accessed 2025 Sep 25.

[26] Nabavizadeh N, Elliott DA, Chen Y et al. Image guided radiation therapy (IGRT) practice patterns and IGRT’s impact on workflow and treatment planning: results from a national survey of American Society for Radiation Oncology members. Int J Radiat Oncol Biol Phys 2016;94:850–7. 10.1016/j.ijrobp.2015.09.035. 26972658

[27] Akino Y, Tohyama N, Akita K et al. Modalities and techniques used for stereotactic radiotherapy, intensity-modulated radiotherapy, and image-guided radiotherapy: a 2018 survey by the Japan Society of Medical Physics. Phys Med 2019;64:182–7. 10.1016/j.ejmp.2019.07.009. 31515018

