# Supplementary material for: Imaging dose in image-guided radiotherapy for localized prostate intensity-modulated radiotherapy: a nationwide survey in Japan
Source: J Radiat Res. 2025 Dec 18;67(1):95–103. doi: 10.1093/jrr/rraf080 (PMC12856032; doi:10.1093/jrr/rraf080)

**Supplementary Tables and Figure**

**Supplementary Table 1.** Imaging parameters of kilovoltage-computed tomography modalities in TomoTherapy systems

| Property | Answer | N (%) |
| --- | --- | --- |
| Scan area | Whole target | 0 (0) |
|  | Partial target | 8 (100) |
| Anatomy | Head | 0 (0) |
|  | Thorax/whole body | 3 (37.5) |
|  | Pelvis | 5 (62.5) |
| Body size | Small | 6 (75.0) |
|  | Medium | 0 (0) |
|  | Large | 2 (25.0) |
|  | Extra-large | 0 (0) |
| FOV | 27 cm | 0 (0) |
|  | 44 cm | 6 (75.0) |
|  | 50 cm | 2 (25.0) |
| Mode | Fine | 2 (25.0) |
|  | Normal | 6 (75.0) |
|  | Coarse | 0 (0) |

**Supplementary Table 2.** Imaging parameters of MV-CT modalities in TomoTherapy systems

| Property | Answer | N (%) |
| --- | --- | --- |
| Scan area | Whole target | 1 (5.0) |
|  | Partial target | 19 (95.0) |
| Pitch (slice thickness) | Fine (1.0 mm) | 0 (0) |
|  | Fine (2.0 mm) | 0 (0) |
|  | Normal (2.0 mm) | 6 (30.0) |
|  | Normal (4.0 mm) | 2 (10.0) |
|  | Coarse (3.0 mm) | 10 (50.0) |
|  | Coarse (6.0 mm) | 2 (10.0) |

**Supplementary Table 3.** Statistics of CTDI_vol_ in kV-CT/CBCT for Varian, Elekta, and Tomotherapy systems

|  | N | Minimum | 25^th^ percentile | 50^th^ percentile | 75^th^ percentile | Maximum | Average |
| --- | --- | --- | --- | --- | --- | --- | --- |
|  |  | [mGy] | | | | | |
| Total | 122 | 0.6 | 6.5 | 11.3 | 16.0 | 29.6 | 12.1 |
| Varian | 89 | 2.0 | 6.6 | 11.7 | 16.0 | 28.1 | 11.8 |
| Elekta | 25 | 0.6 | 4.9 | 11.5 | 22.0 | 29.6 | 13.8 |
| Tomotherapy | 8 | 6.0 | 9.0 | 11.0 | 11.0 | 12.0 | 10.0 |

**Supplementary Figure 1.** Box plots of CTDI_vol_ in kV-CT/CBCT for Varian, Elekta, and Tomotherapy systems. The box represents the interquartile range, and the whiskers indicate the minimum and maximum values within a specific range.


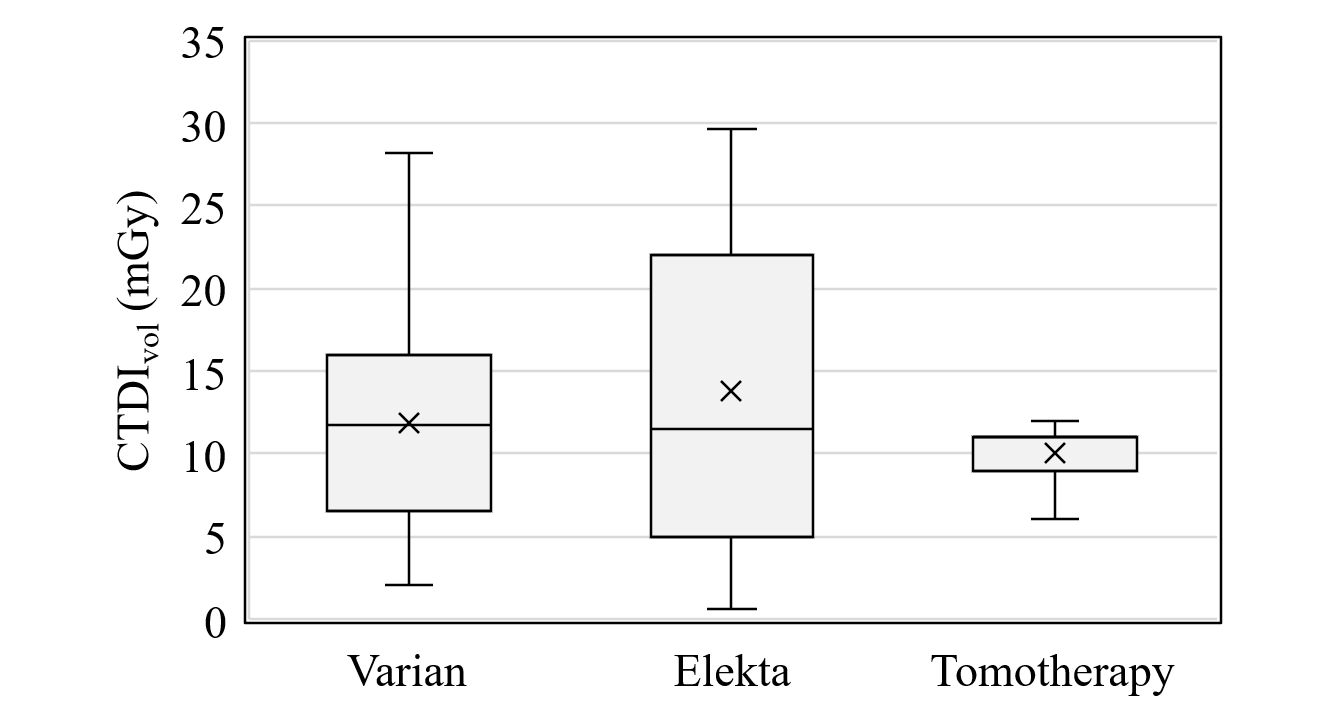

Supplement: 8R_Supplemental_rev1_clean_v20251001a_rraf080 [file 8r_supplemental_rev1_clean_v20251001a_rraf080.docx]
